# Treatment of Epidemic Cholera

**Published:** 1849-08

**Authors:** 


*Treatment of Epidemic Cholera.* By LEWIS SHANKS, M. D., of Memphis, Tennesee.

From a well written article on the "Origin, Progress, Symptoms, and Treatment of Pestilential Cholera, as it occurred in the Mississippi Valley, and especially in the city of Memphis," contained in the *American Journal of the Medical Sciences*, for July, 1849, we quote the portion relating to treatment.

EDITOR BUFFALO MEDICAL JOURNAL.

---

On the treatment of cholera so much has been written, which is accessible to every medical reader, that more than a brief outline of the plan of treatment adopted, and considered most successful here, is unnecessary.

1*st.* *Precursory Stage.*—This stage in its commencement was, doubtless, arrested and cured in many cases by various means—such as brandy, capsicum, laudanum, peppermint, camphor, &c., separately and combined; but after it was fully established, though the use of these means often suspended for a time the purging, it most commonly returned in a more aggravated form, and required other remedies in conjunction with them, to restore and correct the secretions and make the cure permanent. For this purpose, mercurials in moderate quantity, if not indispensable, were most efficient. In the crowd of business, for convenience and dispatch, finding it answer the indications well, I used a pill in this stage made of calomel 3 to 4 grains, camphor 1½ to 2 grains, opium 1 grain, oil of capsicum half a drop, or capsicum 1 grain. These pills to be taken from one to four for the first dose, according to the urgency of the purging, and from one to two to be repeated every hour or two, until the uneasiness or pain in the bowels and diarrhœa are relieved; their effects to be aided by a hot mustard foot-bath, close confinement to the horizontal position in bed, the use of warm, gently stimulating drinks, as sage, balm, ginger, or green tea, or toast, or rice-water, with bicarbonate of soda, and also by hot applications to the feet, and a mush poultice, rendered stimulating by the addition of pepper or mustard applied over the stomach and abdomen. After the purging was arrested, and perspiration restored by these means, if the bowels remained constipated more than twenty-four hours, they were moved by a teaspoonful of turpentine, combined with one or two tablespoonfuls of castor oil, which generally brought off bilious stools. During several days the diet was restricted to gruel, rice, tea and crackers, chicken water, &c., which generally established the cure.

2*d.* *Second Stage.*—Called on to institute treatment in the second stage, when the purging of thin, muddy, or rice water discharges, with vomiting and cramps superadded, indicated the urgency of prompt and efficient relief to prevent the pending and fatal collapse, I required a strict observance of the horizontal position in bed, as free ventilation as possible, to supply fresh air for respiration, and gave, instead of pills, which were too slow in dissolving in the stomach, for this urgent condition, the following compound powder:—calomel, camphor, and capsicum, each, equal quantities, and opium 1 grain to every 20 grains of the compound, all to be well pulverixed and rubbed together. Of this from 10 to 30 grains were given, according to the urgency of the symptoms, at a dose, mixed with from a teaspoonful to a dessertspoonful of laudanum when necessary, and washed down with a small quantity of cold water; and all drinks were prohibited until its impression was made on the stomach and general system. If vomited up, the dose to be repeated, and if the purging and cramps were not relieved, the dose to be repeated every half hour or hour, in such quantities, both of the powder and laudanum, as seemed necessary to control the symptoms. These remedies were aided by the *hot* mustard foot-bath, hot applications to the feet and back, a large blistering plaster applied over the stomach, &c. As soon as these means controlled the irritability of the stomach, warm drinks, toddy, toast water or teas, were allowed ad libitum. But while the stomach continues irritable, drinks of all kinds are almost immediately vomited up, and the thirst and vomiting are both sooner relieved when drinks are prohibited, and small lumps of ice only are allowed.

The compound powder and the laudanum were given in quantities proportioned to the age, constitution, and ability of the patient to bear them, and the emergency of the case. When the serous discharges from the bowels were copious, though the number of stools may not have been numerous, with a tendency to speedy collapse, especially when accompanied with violent cramps, a large dose both of the capsicum powder, and in some cases 20 to 30 grains of calomel additional, and the laudanum were given, repeated with or without the additional calomel, at short intervals, if necessary to arrest the symptoms. At this critical juncture, when the physician is often first called, the life of the patient depends upon a correct judgment being formed of the *dose* which the patient can bear with safety; and the *dose* necessary should be given at once, and if it does not produce the effect desired in from three quarters to an hour, it must be repeated until the object desired is attained. The propriety and necessity of this prompt and bold practice when the treatment is commenced under these circumstances are obvious—for one more stool—the delay or loss by small and inefficient *doses* of one hour, may bring about fatal collapse. In addition to the other means, an enema of a gill of starch and a drachm or two of laudanum, or, what was more efficient and better retained, one to two drachms of laudanum with one drachm of spirit of camphor, ether, and aromatic spirit of ammonia, or essence of peppermint, each, combined with the starch, is a valuable auxiliary, both in arresting the discharges and rousing up and sustaining the nervous energies of the system.

These means being successful in arresting the symptoms, and producing reaction, perspiration, and healthy secretions and excretions, the bowels were generally moved in due time, and the discharges were bilious; but if the bowels continued locked up beyond twenty-four or thirty-six hours, castor oil and turpentine were administered to open them. These results being produced, mild diluent, and when necessary, gently stimulating drinks, and abstemious diet, generally perfected the case in a few days.

3*d*. *Collapsed Stage.*—In the collapsed stage the same course was pursued, with the addition of twenty to forty grains of calomel to each dose of the compound powder. The additional large doses of calomel were given, not so much with a view to its direct effect in producing reaction, as to its well-established effects in allaying irritability of the stomach, arresting watery discharges from the bowels, and restoring secretion and excretion when reaction was produced by other means, and thus preventing consecutive fever. By the use of calomel in this way, when its wonted effect upon the secretions was produced, consecutive fever was invariably prevented, as the result of either the second or third stage of the disease; and the only troublesome secondary symptom to the patient, which occasionally occurred, was ptyalism. Indeed, this specific effect of mercury, in any given ratio of doses and time, seemed more liable speedily to be produced in cholera when full reaction took place, than in any other form of acute disease.

In a case of speedy recovery in a man about twenty-five years old, from a state of pulseless and apparently hopeless collapse, attended with violent cramps, I gave, as a first dose, about twenty-five grains of the compound capsicum powder, with thirty grains additional of calomel, mixed in a large tablespoonful of laudanum, washed down with a glass of brandy. In a few minutes it was vomited, by a spasmodic action, far across the room. The same dose was repeated, and washed down by a swallow of cold water. The powder and laudanum, in the same quantity, were twice more repeated in the space of an hour and a half, and no drinks allowed until the stomach was settled, and partial reaction indicated absorption, when diluent drinks, with a moderate quantity of brandy, were given freely. In addition to the other means already mentioned in this and many other cases, a prompt and important effect was produced by woollen cloths wrung out of nearly boiling *hot* water, and applied to the stomach, spine, and extremities. After the three doses, laudanum alone was given, in teaspoonful doses, at intervals of two to four hours, until the cramps were relieved. In eighteen hours, this man took from an ounce and a half to two ounces of laudanum. The next day he was much better, though he had slept but little, and evinced no decided effect from the laudanum, except itching of his nose and of the surface generally. The following day he was well enough to sit up, and recovered speedily, with but a slight affection of the gums from the calomel.

So large an amount of opium was not given in any other case. The result, however, proved that, though it was retained in the stomach, and absorption was re-established in a few hours, still the feeble state of the circulation, and, for a time, the want of supply of the healthy and necessary amount of arterialized blood in the brain, prevented the ordinary effects of the opiate from being produced on the brain and nervous system.

Being fully convinced, from many years of careful observation, of the importance and necessity of the free use of opiates and calomel in congestive fever, of the continued form,—from the pathological likeness of cholera, the same practice to some extent was adopted.

In cases of congestive fever, coming on as they frequently did here, eight or ten years since, with lassitude, slight chilliness, sick stomach, vomiting, prostration, &c.: vomiting and thirst increasing in the same ratio with each other; the pulse waning; the surface and extremities becoming cold, with increasing complaint of oppression and internal heat; though in some few cases this complaint of heat was absent, and the tongue and breath were cool;—the pulse gradually diminishing down to a thread; the surface wet with cold, clammy perspiration; the skin shrivelled and sodden, as in cholera, but never so blue and marbled; the mind generally clear; but during the development of this category of symptoms, which required from three to five days to arrive at their acme, without any evidence of paroxysms, except an increase of oppression, frequency and feebleness of the pulse in the evenings, there were almost invariably great torpor and obstinate constipation of the bowels.

In these cases, opiates were tolerated and required in large doses; and my practice has been to administer them in combination with calomel, so as to give ten to twenty grains of the latter three to four times a day, with as much of the former as was necessary to relieve the concentration of nervous irritation upon the stomach, and to sustain the innervation of the brain and general system, until the action of the nervous and vascular systems were equalized by the influence of the opiates, and other appropriate means, and permanent healthy action secured by the general alterative and stimulating effects of the mercury upon the secretory and excretory organs.

This practice has been so uniformly successful in the condition of the system described, that I have seen no reason to change it for any other; though to make it so, in extreme cases, an amount of opiates equal to twenty grains of the gum in twenty-four hours might be required.

In cases of extreme irritability of the stomach in congestive fever, when the internal use of opiates failed to control it, and the general restlessness, after blistering the precordial region, the external application of morphia acted promptly and efficiently. This is more the case in cholera, and is a means of more value, when there are great depression and restlessness, and the stomach continues to be irritable, and no absorption is taking place from the stomach and bowels, than almost any other.

In the collapsed state of congestive fever, ice rubbed over the extremities and the parts to which it is applied wiped dry, and well rubbed, and this followed by external heat, to be repeated every half hour or every hour, is a valuable means of restoring excitability and producing general warmth and reaction. This remedy used in cholera, when the system was not too much drained, and the blood rendered too thick to be invited back into the superficial capillaries, is decidedly valuable. But in the collapsed stage, when the blood has retired from the extremities and surface, it has failed to do more than temporarily improve the color of the skin, and revive for a transient space of time the slight evidences of circulation that still existed. Like all other means in that condition, generally it fails in producing any permanent benefit.

No other disease, except sporadic cholera morbus, presents so many resemblances to epidemic cholera, as this continued form of congestive fever, in its symptoms, pathology and treatment; though in it the profuse purging and cramps characteristic of cholera are wanting, and indeed in most cases the bowels are torpid; yet the general likeness is often striking, and, compared with cases described by Mr. Thorn and others, on the Ganges, where cholera collapse and death sometimes occur without purging, the resemblance is still greater.

Now this form of congestive fever is clearly the effect of malarial influences, and it is peculiar to low, damp, and malarious regions. The specific virus of cholera tends universally to affect the bowels and produce watery passages and cramps, and thus to constitute striking diagnostic symptoms between it and the most malignant forms of malarial fever. There seems to be no good reason, however, to *disbelieve* that the *cholera virus* and *malaria* may *co-exist* and *conjointly* act upon the system; and, indeed, the fact that the cases of cholera which result speedily in collapse, without any purging, thus resembling pernicious fever, are only reported by physicians who have witnessed the disease in highly malarious and hot regions, such as the Delta of the Ganges, strongly corroborates their *conjoint* effects upon the system, and the occasional *predominance* of the malarial influence, in the production of such cases.

The ataxic cases which occurred here during the cold dry weather, were attended with pain in the head, stomach, and bowels, and prostration, without much, and in some cases without any purging; the pulse slow, 40 to 60, and soft; the surface cool, and the extremities cold and cramped.

Opiates in these cases, and especially when the cephalalgia was severe, could only be used with great caution. The abstraction of blood in this condition, and in this form of the disease only, seemed indicated and useful to develop reaction, and aid in equalizing the circulation and restoring healthy secretion.

*4th. Consecutive Stage.*—When the plan of treatment I have given was adopted and properly carried out, in the previous, or three stages proper to cholera, consecutive fever rarely occurred; but treated exclusively with anodynes, astringents, and stimulants, the recoveries were more apt to be slow, and attended with want of proper secretion and with gastro-enteritic inflammation, presenting a more or less protracted case of a typhoid form of fever. The treatment of this condition requires the use of alteratives, diluents, diaphoretics, &c.; a careful attention to the condition of the brain, to prevent a determination of disease to that organ.

The importance and effect of a few of the prominent remedies in the treatment of cholera, deserves a passing notice.

*Opium and Calomel.*—Much importance has been attached to the free use of opium and calomel, especially in the second and third stages of cholera. While, in the precursory stage, they are important to moderate and arrest the purging, and at the same time to correct the morbid secretions, in the second and third stages the life of the patient depends upon the attainment of these objects in the shortest possible time.

To effect these objects, both observation and analogy prove that no other remedies are so efficient and certain, as opium and calomel. The want of ordinary susceptibility of the nervous system to the impression of remedies; the suspension of absorption from the stomach and bowels; the regurgitation of the chyle, and the rapid pouring into the stomach and bowels of the *liquor sanguinis* from the general system, carrying out speedily all the remedies given either by vomiting or purging, and by this current of determination to the bowels preventing their absorption and diffusion through the system; all show the indispensable necessity of large doses to secure the desired object.

The opiates—and *laudanum* or *morphia*, from their more prompt action, are best—must be given in large doses, sufficient to arrest the discharges. Other effects, not much less important in this condition, are more certainly produced by *it* than any other remedy. It sustains the nervous energies and the action of the heart; it relieves local irritation and cramps, and equalizes innervation, and thus helps the system to rally under the depressing influence of the morbid poison. For the additional effort, of safely eliminating the poison from the system, and correcting the morbid secretions produced by its influence, the great weight of medical authority, and my own exprience, are in favor of calomel above all other remedies. It must, however, be given in large doses, and immediately with the laudanum, so that no time may be lost in securing its alterative action. To the inexperienced, the use of calomel in large doses, and immediately with the laudanum, so that no time may be lost in securing its alterative action. To the inexperienced, the use of calomel in large doses, when there is copious serous purging, might seem a paradox; but who that has almost invariably found a full dose of calomel, uncombined with opium, arrest the frequent watery discharges in ordinary diarrhœa, and gradually give them healthy consistence, could doubt the propriety and importance of its use in this condition, with a view to its alterative effects?

Large doses of opiates are warranted, and proved to be safe in this condition of the system, not only by ample experience, but from analogy. If the case terminates fatally, nothing is certainly determined as to the relative effects of the remedies used, and the disease upon the system; because, when it is exhausted by the discharges, the general enervation results in insensibility and death, without the use of opiates; this insensibility and stupor which immediately precedes death may be attributed incorrectly to the effects of the opiates.

But when a violent case is treated by the free use of opiates, such as the case of complete collapse reported, then, and then only can the favorable, or unfavorable effects of this remedy be fairly and fully determined, by the clear manifestation of its effects.

In cases of feeble action of the heart, and deficient supply of blood to the brain, either from hemorrhage or other causes, large doses of opiates are not only safely tolerated, but required to sustain the system, as in uterine hemorrhage, mania-à-potu, congestive fever, &c.

For the purpose of stimulating and constringing the exhausted surface of the stomach and bowels, and also rousing up and sustaining the depressed nervous energies of the system, and producing reaction, capsicum, camphor, spirit of ammonia, brandy, and various other stimulants and aromatics are valuable auxiliaries.

Another very important requisite deserves to be more particularly noticed in the successful treatment of cholera, after the system is at all exhausted by the discharges of its white blood; absolute confinement to the *horizontal* position in bed.

In no condition except after profuse hemorrhage, is the effect of the erect posture so immediately manifest in producing faintness, sick stomach, enfeebled pulse, &c., as in cholera. In a state of partial reaction, and a favorable and promising change in the symptoms, after great exhaustion from profuse discharges, I have seen all undone, and the fatal collapse reproduced by the patient's assuming the erect posture on getting out of bed.

